# Metabolic Predictors of CAD: Focus on Cystine, Methionine, Proline, and Threonine Circulating Levels—Exploratory Pilot Study

**DOI:** 10.3390/jcm14238356

**Published:** 2025-11-25

**Authors:** Tomasz Urbanowicz, Dagmara Pietkiewicz, Szymon Plewa, Beata Krasińska, Ievgen Spasenenko, Katarzyna Gabriel, Karolina Jezierska, Zbigniew Krasiński, Mariusz Kowalewski, Jan Matysiak, Andrzej Tykarski

**Affiliations:** 1 Cardiac Surgery and Transplantology Department, Poznan University of Medical Sciences, 61-848 Poznan, Poland; 2 Department of Inorganic and Analytical Chemistry, Faculty of Pharmacy, Poznan University of Medical Sciences, 61-614 Poznan, Poland; 3 Department of Hypertensiology, Angiology and Internal Medicine, Poznan University of Medical Sciences, 61-848 Poznan, Poland; 4 Moles Diagnostic Center, 10/1 Ogrodowa Street, 61-821 Poznan, Poland; 5 Department of Vascular, Endovascular Surgery, Angiology and Phlebology, Poznan University of Medical Science, 61-701 Poznan, Poland; 6 Clinical Department of Cardiac Surgery and Transplantology, Central Clinical Hospital of the National Medical Institute of the Ministry of Interior and Administration, 02-507 Warsaw, Poland

**Keywords:** coronary disease, CAD, threonine, cystine, threonine, proline, methionine, biomarkers, amino acids

## Abstract

**Background**: Coronary disease (CAD) is a multifactorial complex pathology characterized by excessive inflammatory activation and oxidative stress. Amino acids are among the potential biomarkers for cardiovascular pathology. The analysis aimed to investigate the possible relationship between proteomic profiling and coronary artery disease risk as novel markers of CAD. **Methods**: Patients with similar demographic and clinical profiles, including the prevalence of comorbidities such as arterial hypertension, dyslipidemia, and diabetes mellitus, were divided into two groups based on the results of their coronary angiograms. Serum amino acid levels were measured using liquid chromatography–tandem mass spectrometry. **Results**: Patients with significant coronary atherosclerosis confirmed in coronary angiograms were characterized by higher levels of circulating cystine, threonine, methionine, and proline. The number of involved coronary arteries in atherosclerotic processes revealed a correlation with circulating levels of threonine, methionine, and proline, not cystine. The multivariable logistic regression analysis for any significant coronary artery disease prediction revealed higher values of circulating threonine as a possible risk factor. Thereafter, a subanalysis was conducted to examine the relationship between amino acid levels and atherosclerotic risk in specific coronary arteries. The multivariate analysis revealed cystine and proline as potential risk factors for atherosclerosis of the left descending artery (LAD). Higher values of threonine were identified as a possible risk factor for atherosclerotic plaque location in the circumflex artery in multivariate regression analysis. Proline circulating levels were found to be prognostic for right coronary artery disease. **Conclusions**: Elevated circulating levels of amino acids, including cystine, threonine, methionine, and proline, were observed in patients with significant coronary artery disease in our exploratory pilot study. The high circulating amino acid levels can be predictive of coronary artery disease in our multivariate models.

## 1. Introduction

Atherosclerosis results from interactions between traditional modifiable and non-modifiable risk factors [1]. Arterial hypertension, dyslipidemia, and diabetes mellitus are among the most prominent modifiable risk factors, accompanied by smoking, obesity, sedentary lifestyle, and dietary routines [2]. Coronary artery disease (CAD) remains a significant epidemiological health challenge. However, traditional risk factors have not only been well-established but also their satisfactory control in epidemiological studies has been confirmed [3]. They are believed to be involved in the fundamental processes of atherogenesis, causing chronic vascular inflammation and endothelial dysfunction, which results in lipid accumulation and atherosclerotic plaque formation [4]. Geographic disparities in the prevalence of coronary artery disease have been reported [5]. The urbanization characterized by high-income countries is postulated as a possible non-traditional risk factor [6].

Increasing age, male sex, and a positive family history of cardiovascular events are regarded as strong non-modifiable CAD risk factors [7]. The psychosocial stress and co-existence of chronic kidney disease, or inflammatory and autoimmune disorders, have been postulated as significant CAD risk contributors [8].

The role of inflammatory activation in the progression of atherosclerosis and acute coronary syndromes is postulated. The plaque cap heterogeneity is secondary to immunological components, involving extracellular matrix degradation [9]. Macrophages, one of the key players in atherosclerotic lesions, induce numerous immune and non-immune pathways and can polarize into pro- and anti-inflammatory phenotypes [10].

In recent times, the potential role of metabolic biomarkers in atherosclerosis, including metabolomic profiling [11], has been explored. Zhang et al. [12] in their analysis pointed out the possible inverse relation between circulating amino acids and diastolic dysfunction. Several studies have demonstrated a relationship between the serum concentration of specific amino acids and the prevalence of increased atherosclerosis, including branched-chain and aromatic amino acids. The potential impact of elevated circulating levels of branched-chain amino acids on insulin resistance was noted, suggesting their involvement in insulin signaling pathways that may promote chronic inflammation and contribute to endothelial dysfunction. In previous studies [13], the role of amino acid-derived metabolites, such as asymmetric dimethylarginine, in CAD risk was postulated. It acts as an endogenous inhibitor of nitric oxide synthase. The possible relationship between serum amino acid concentration and gut microbiota has also been recognized as a potential modulator of CAD, underscoring the complexity of metabolic mechanisms underlying CAD.

Based on previous results, incorporating serum amino acid analysis into CAD risk assessment strategies may offer novel possibilities for personalized prevention and early detection, followed by targeted therapeutic opportunities. The prospective study aimed to investigate the possible association between circulating amino acid levels and coronary artery risk confirmed by coronary angiograms in patients presenting with chronic ischemic disease.

## 2. Materials and Methods

Out of 54 (33 (61%) male) consecutive patients diagnosed with chronic coronary syndrome, who were admitted for planned coronary angiography by their cardiologist. The 27 (18 (67%) men) were diagnosed with significant coronary disease (CAD group) and compared with 27 (15 (56%) men) patients with normal angiograms (non-CAD group).

Inclusion criteria included consecutive patients diagnosed with stable chronic coronary syndrome classified as angina equivalent diagnosed in the Internal and Hypertension Department in 2024 who a cardiologist for control angiography referred due to angina equivalent. Patients presenting with easy fatiguability, exercise intolerance, and shortness of breath on exertion were included. Demographical data (age, sex, body mass index (BMI), followed by clinical characteristics such as arterial hypertension, diabetes. Mellitus, dyslipidemia, chronic obstructive pulmonary disease, and kidney dysfunction (defined as glomerular filtration rate below 60 mL/kg/m^2^) were analyzed, followed by familial cardiovascular disease history and nicotine addiction (active and former). The analysis involved patients presenting with coronary disease in the main arteries (LAD, Cx, and RCA), excluding left main coronary disease (assigned to unstable disease patients). Patients presenting with coronary disease in the diagonal and marginal branches were excluded from the analysis. The coronary artery disease group comprised patients with coronary lumen stenosis of at least 50%. On the contrary, patients with normal coronary angiograms were enrolled in the non-CAD group. Patients with coronary artery occlusion or hemodynamically insignificant atherosclerotic coronary lumen narrowing (10–49%) were excluded from the study.

Upon admission, laboratory tests and an echocardiographic examination were performed by an experienced echocardiographer. Laboratory tests obtained from peripheral blood analysis after twelve hours of fasting included whole blood count analysis (white cell count, hemoglobin and hemoglobin concentration, and red cell distribution width), kidney function tests (serum creatinine concentration and glomerular filtration rate), liver function tests (alanine aminotransferase), lipoprotein (a) serum concentration, lipidogram (total cholesterol, low- and high-density lipoprotein), and triglycerides) followed by NT–pro–natriuretic peptide concentration. Transthoracic echocardiographic parameters included left ventricular diastolic diameter (LVED), intraventricular septum (IVS), global longitudinal strain (GLS), and left ventricular ejection fraction (LVEF). The aortic/mitral and tricuspid valves’ function and morphology were examined.

Coronary angiography was then performed at a high-volume hemodynamic cardiology team. An experienced hemodynamic team evaluated the results of coronary angiography, and significant coronary disease was defined as coronary lumen narrowing of at least 50% [14].

The exclusion criteria included acute coronary syndromes, significant valve pathologies, or any special diet restrictions within 12 months preceding the admission.

### 2.1. Amino Acids Analysis

A twelve-hour fasting period preceded all blood sample collection, which was performed on admission. The blood samples were centrifuged for five minutes, and serum aliquots were stored at −80 °C until analysis. The quantitative determination of amino acids was performed using liquid chromatography coupled with tandem mass spectrometry (LC-MS/MS) with the MassChrom Amino Acids Analysis kit (Chromsystems Instruments & Chemicals GmbH, Germany).

For sample preparation, 25 µL of serum was mixed with 50 µL of reconstituted internal standard solution and 400 µL of precipitation reagent. Samples were vortexed for 30 s and then centrifuged at 16,000× *g* for 5 min. The supernatant was transferred to autosampler vials. Liofilized calibration standards and quality control (QC) samples were reconstituted according to the manufacturer’s manual and prepared in the same way as the serum samples.

All samples were randomized before analysis. A triple quadrupole tandem mass spectrometer 5500+ QTRAP (Sciex, Framingham, MA, USA) and a high-performance liquid chromatograph 1260 Infinity II (Agilent Technologies, Santa Clara, CA, USA) were utilized. Chromatographic separation was performed using a binary gradient on the MassChrom analytical column, with Mobile Phase A and Mobile Phase B. The LC gradient was: 0–0.5 min, 100% A; 1 min, 89% A; 6.3 min, 89% A; 12.3 min, 83% A; 14.8 min, 68% A; 14.9–17.4 min, 0% A (100% B); re-equilibration to 100% A by 19.3 min. The flow rate was adjusted accordingly (0.8–1.8 mL/min). The column oven temperature was maintained at 25 °C. The injection volume was set at up to 5 µL. Data acquisition was performed using Analyst software (AB Sciex, v. 1.7.3), with retention time adjustments performed before final quantification to ensure peak accuracy. The mass spectrometer operated in positive electrospray ionization mode (ESI+) with multiple reaction monitoring (MRM) transitions specific for each amino acid. QC samples were run at intervals within each sequence to monitor accuracy and reproducibility. Peak integration, data processing, and quantification of all analytes were performed using SciexOS software (AB Sciex, v. 3.3.0.12027). All the measured analytes passed quality control. The intra-assay coefficient of variation was calculated from the QC sample repetitions and was 4.98%.

### 2.2. Statistical Analysis

The continuous variables were reported as medians and interquartile ranges (Q1–Q3) if the data did not follow the normal distribution. Categorical data were presented as numbers and percentages.

The sample size was estimated using the minimum sample size estimation tool in the PQStat software (version 1.8.6). Based on the assumption of an undetermined target population size, α = 0.05, and β = 0.2, the minimum sample size was estimated at 24 participants. Hence, the authors have decided to collect complete data from at least 25 participants, as well as a roughly equivalent number of controls.

The Mann–Whitney test was used to compare interval parameters between the analyzed groups. Categorical data were compared using a chi-square test of independence. Multivariable models were used to predict the risk of coronary artery disease, including the location of atherosclerosis in specific arteries. The multivariable model was assessed using the best subset method. The obtained results were presented as odds ratios (ORs) and 95% confidence intervals (95% CIs). The receiver operating characteristic (ROC) curve was used to evaluate the accuracy of confirming multivariable analysis results in the prediction model. Statistical analysis was performed using JASP version 0.14.1 (University of Amsterdam, Netherlands), with a significance level set at *p* < 0.05 (https://jasp-stats.org, accessed on 16 December 2020).

## 3. Results

The CAD group comprised 27 patients, including 18 males with a median age of 66 (62–72). There were 27 more patients (15 males) with a median age of 68 (61–72) years in the non-CAD group. There were no significant differences regarding age (*p* = 0.633), sex (*p* = 0.413), BMI (*p* = 0.242), followed by co-morbidities such as arterial hypertension (*p* = 0.893), diabetes mellitus (*p* = 0.155), dyslipidemia (*p* = 0.142), chronic obstructive pulmonary disease (*p* = 0.582), and kidney dysfunction defined as glomerular filtration rate below 60 mL/kg/m^2^ (*p* = 0.224) between both group. The family CAD history was insignificant between the analyzed subpopulations (*p* = 0.233). Detailed information is presented in Table 1.

### 3.1. Laboratory Results

The whole blood count analysis revealed significant differences in red cell distribution width (RDW) (*p* = 0.032). The glomerular filtration rate was indifferent between the groups (*p* = 0.144), as was the liver function test (*p* = 0.823). Neither lipoprotein (a) (*p* = 0.424) nor N-terminal pro-brain natriuretic peptide (*p* = 0.352) distinguished the analyzed groups. The detailed information was included in Table 2.

### 3.2. Echocardiography

There were no significant differences between CAD and non-CAD groups regarding echocardiographic parameters including: left ventricular diastolic diameter (LVED) and intraventricular septum (IVS) reaching the value of 52 (46—55) mm vs. 50 (47–57) mm (*p* = 1.000), and 11 (10–12) mm vs. 11 (10–12) mm (*p* = 0.992), respectively. The left ventricular performance was evaluated by global longitudinal strains (GLS) and left ventricular ejection fraction (LVEF) estimation. There were no differences regarding GLS (13.0 (10.5–17.8) vs. 13.0 (8.00–15.5), (*p* = 0.878)), nor LVEF (49 (39—57)% vs. 49 (38–58)% (*p* = 0.841)) between CAD and non-CAD groups.

### 3.3. Coronary Angiography

The coronary angiography confirmed significant coronary atherosclerotic lesions in 27 patients enrolled in the prospective analysis. The median number of involved arteries was 1.5 (range, 1.0–3.0). Single-, two-, and three-vessel disease were noted in 12 (44%), 6 (22%), and 9 (33%) patients, respectively.

The significant coronary artery disease, defined as lumen narrowing at least 50% was noted in the left descending artery in 21 CAD patients (78% of the 27 participants). Significant stenosis of the circumflex artery and the right coronary artery was revealed in 16 (59%, out of 27 CAD group) and 10 (27%, from 27 CAD group) patients, respectively.

### 3.4. Amino Acid Concentration

For each trace element analysis, we performed three calculations (Cal 1, 2, and 3) and estimated quartile concentrations (QC 1, 2, and 3). The results were presented as median (Q1–Q3) concentrations in Table 3.

**Figure 1:**
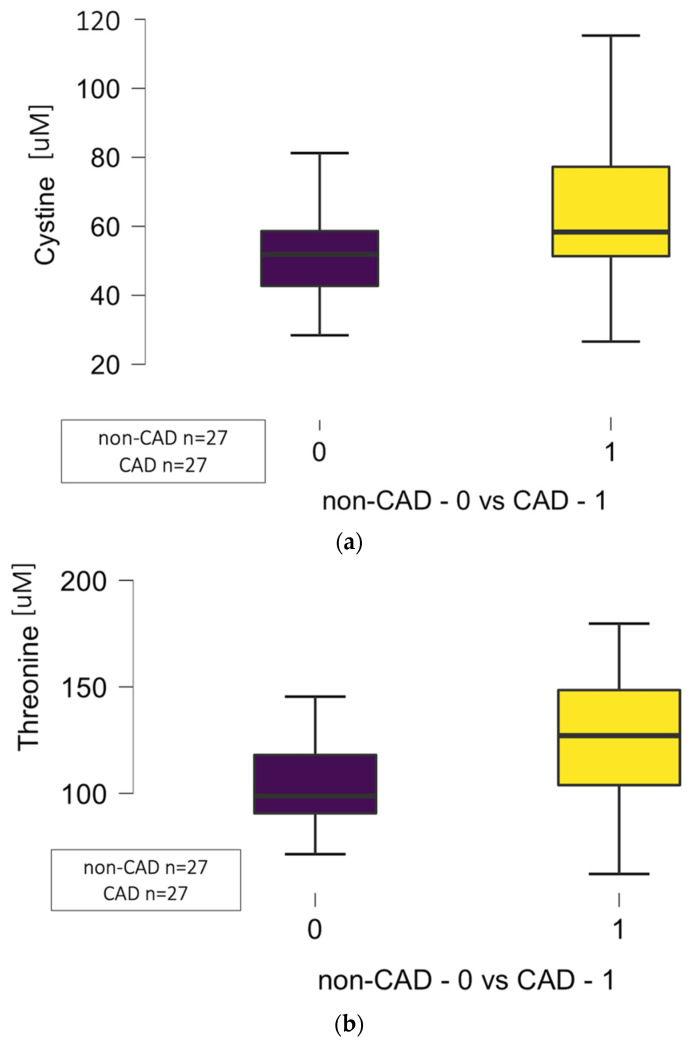

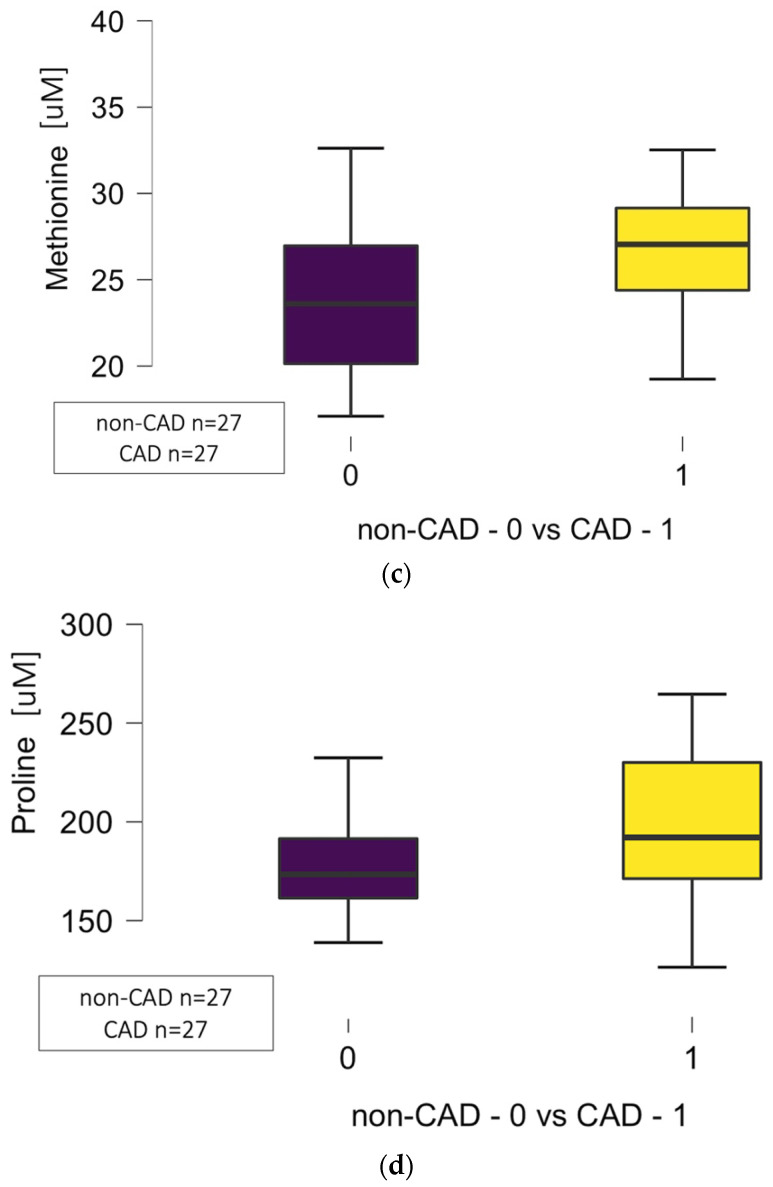
(**a**) Differences in circulating levels of cystine in non-CAD (0) vs. CAD (1) groups (*p* = 0.036). (**b**) Differences in circulating levels of threonine in non-CAD (0) vs. CAD (1) groups (*p* = 0.006). (**c**) Differences in circulating levels of methionine in non-CAD (0) vs. CAD (1) groups (*p* = 0.013). (**d**) Differences in circulating levels of proline in non-CAD (0) vs. CAD (1) groups (*p* = 0.042) related to atherosclerosis in the left descending artery (LAD). Abbreviations: CAD—coronary artery disease, μM—micromole.

The significant differences in circulating amino acid concentrations regarding cystine (*p* = 0.036), threonine (*p* = 0.006), methionine (*p* = 0.013), and proline (*p* = 0.042) between CAD (27 patients) and non-CAD (27 patients) groups were noted as presented in Figure 1a–d.

#### 3.4.1. Correlations Between Circulating Amino Acids and the Number of Diseased Arteries

The Spearman correlation between the number of involved coronary arteries in atherosclerotic processes and circulating levels of threonine (r = 0.399, *p* = 0.004), methionine (r = 0.307, *p* = 0.030), and proline (r = 0.412, *p* = 0.003) was noted, but not cystine (r = 0.246, *p* = 0.084).

#### 3.4.2. Certain Amino Acid Concentration and the Particular Coronary Arteries’ Atherosclerosis

##### Left Descending Artery

**Figure 2:**
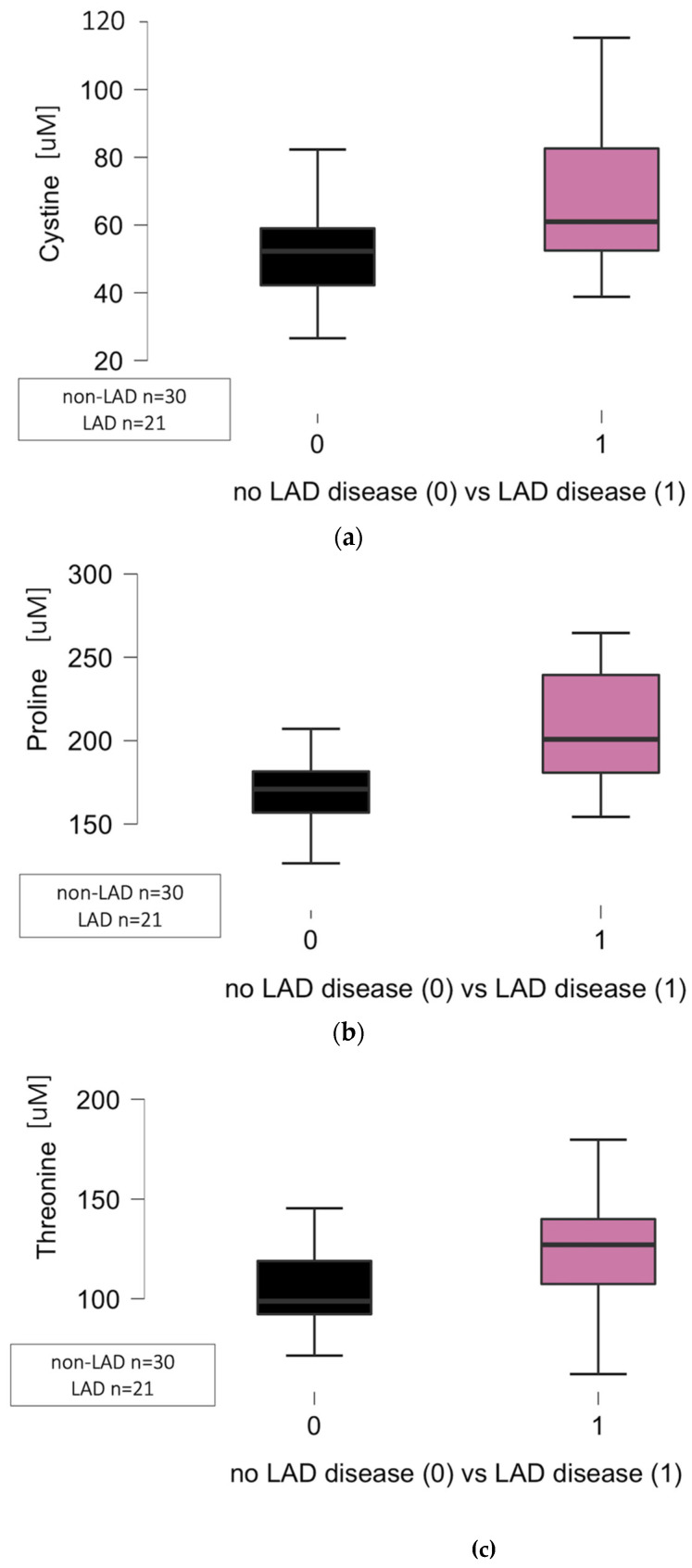
(**a**) Significant differences regarding the cystine circulating levels related to atherosclerosis in the left descending artery (LAD) in the CAD group (1) when compared to the control group (0) (*p* = 0.014). (**b**) Significant differences regarding the proline circulating levels related to atherosclerosis in the left descending artery (LAD) in the CAD group (1) when compared to the control group (0) (*p* = 0.001). Abbreviations: CAD—coronary artery disease, LAD—left descending artery, uM—micromole. (**c**) Significant differences regarding the threonine circulating levels related to atherosclerosis in the left descending artery (LAD) in the CAD group (1) when compared to the control group (0) (*p* = 0.008).

The significant differences regarding the circulating levels of cystine (*p* = 0.014), proline (*p* = 0.001), and threonine (*p* = 0.008), but not methionine (*p* = 0.068), were noted in relation to significant atherosclerotic lesions (at least 50% lumen narrowing) located in the left descending artery (LAD) as presented in Figure 2. The significant atherosclerotic lesions were noted in 21 patients in the left descending coronary artery (CAD group). No atherosclerotic plaques were pointed out in the coronary angiograms of 30 patients, including 27 from the non-CAD group and three from the CAD group.

##### Circumflex Artery

**Figure 3:**
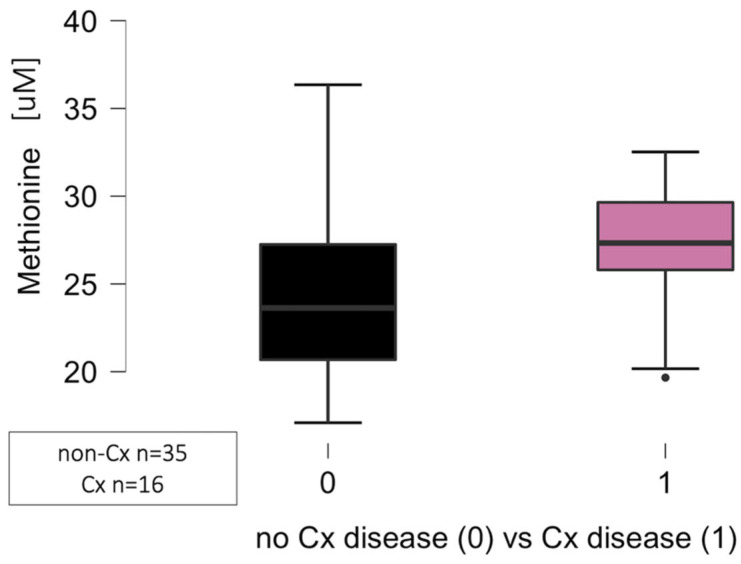
Significant differences regarding the methionine circulating levels related to atherosclerosis in the circumflex artery (Cx) in the CAD group (1) when compared to the control group (0) (*p* = 0.013). Abbreviations: CAD—coronary artery disease, Cx—circumflex artery, uM—micromole.

The significant differences regarding the circulating levels of methionine (*p* = 0.013) but not cystine (*p* = 0.680), proline (*p* = 0.216), and threonine (*p* = 0.066) were noted in relation to the circumflex artery (Cx) disease, defined as lumen narrowing at least 50% when compared to normal angiograms as presented in Figure 3. The analysis was performed in 16 patients with angiographically proven atherosclerotic lesions in the CAD group, compared to 35 patients, comprising 27 patients from the non-CAD group and eight patients from the CAD group.

##### Right Coronary Artery

**Figure 4:**
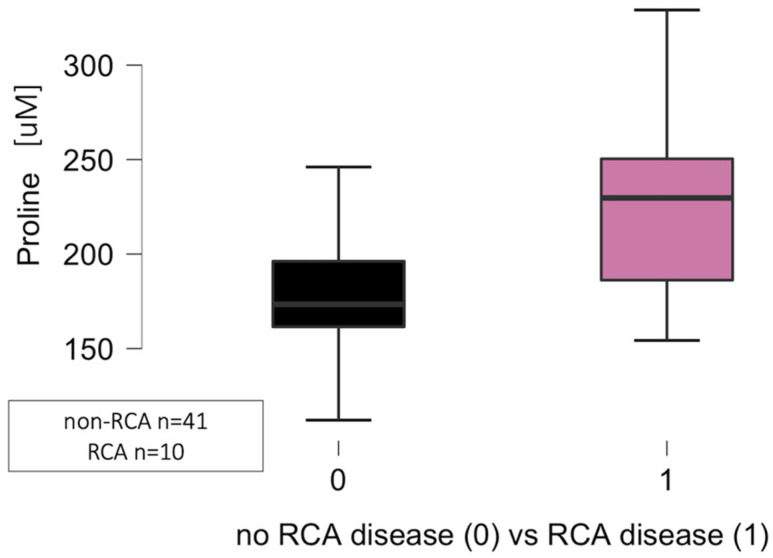
Significant differences regarding the proline circulating levels related to atherosclerosis in the right coronary artery (RCA) in the CAD group (1) when compared to the control group (0) (*p* = 0.006). Abbreviations: CAD—coronary artery disease, RCA—right coronary artery, uM—micromole.

The significant differences regarding the circulating levels of proline (*p* = 0.006), but not cystine (*p* = 0.095), threonine (*p* = 0.052), and methionine (*p* = 0.280) were noted in relation to atherosclerotic plaque location in the right coronary artery (RCA) as presented in Figure 4. The comparison was made between 10 patients with significant RCA disease (lumen narrowing at least 50% in coronary angiogram) and a control group of 41 patients (27 from the non-CAD group and 14 from the CAD group).

#### 3.4.3. Logistic Regression Analysis for Coronary Disease Prediction

##### Any Significant Coronary Disease

The multivariable model was created based on the following parameters: demographic (age, sex, BMI > 30), clinical (arterial hypertension, diabetes mellitus, dyslipidemia, family history), followed by RDW (the only parameter from laboratory results that differentiated the groups) and four amino acids (cysteine, threonine, methionine, and proline) that distinguished both groups.

The multivariable logistic regression analysis for predicting significant coronary artery disease revealed higher values of circulating threonine as a possible risk factor (OR: 1.05, 95% CI: 1.01–1.09, *p* = 0.014). The area under the curve was 0.807, yielding a specificity of 77.8% 64.7% and a sensitivity of 64.7%. The 50% and 75% probabilities were associated with the threonine serum concentrations of 109 and 146, respectively.

##### Left Descending Artery Disease

The multivariable analysis revealed cystine (OR: 1.06, 95% CI: 1.01–1.12, *p* = 0.012) and proline (OR: 1.03, 95% CI: 1.01–1.06, *p* = 0.017) as potential risk factors for atherosclerosis locations in LAD. The receiver operating characteristic (ROC) curve was characterized by an area under the curve (AUC) of 0.876, yielding a sensitivity of 66.7% and a specificity of 86.4%. The 50% and 75% probabilities were associated with the cystine serum concentration of 69 and 183, respectively. The 50% and 75% probabilities were associated with the proline serum concentrations of 214 and 249, respectively.

##### Circumflex Artery Disease

The multivariable analysis for predicting circumflex artery disease revealed higher values of threonine (OR: 1.03, 95% CI: 1.01–1.06, *p* = 0.018), indicating a potential atherosclerotic location in the circumflex artery. The receiver operating characteristic (ROC) curve was characterized by an area under the curve (AUC) of 0.685, yielding a sensitivity of 35.7% and a specificity of 96.9%. The 50% and 75% probabilities were associated with the cystine serum concentrations of 154 and 181, respectively.

##### Right Coronary Artery Disease

The multivariable analysis for predicting right coronary artery disease revealed higher values of proline (OR: 1.03, 95% CI: 1.01–1.05, *p* = 0.007) as a potential risk factor. The receiver operating characteristic (ROC) curve was characterized by an area under the curve (AUC) of 0.787, yielding a sensitivity of 40.0% and a specificity of 94.4%. The 50% and 75% probabilities were associated with the cystine serum concentrations of 242 and 268, respectively.

## 4. Discussion

We present the significant differences in circulating amino acid concentrations in coronary artery disease. Metabolic profiling plays multifaceted roles in the pathophysiology of atherosclerosis, and amino acids are involved in regulating vascular function, activating inflammatory processes, and modulating immune responses. Our results, based on two comparable groups in terms of demographic and clinical characteristics, as well as laboratory and echocardiographic results, indicate significant differences in circulating amino acids in the CAD group.

In patients with coronary artery disease, we observed higher circulating levels of cystine, methionine, proline, and threonine. Our analysis revealed a correlation between specific coronary arteries and circulating amino acids. The left descending artery atherosclerotic involvement was characterized by higher circulating concentrations of cystine, proline, and threonine. The significantly higher circulating levels of methionine were noted in relation to atherosclerosis of the Cx. The higher proline levels were characterized in patients presenting with RCA disease. A previous report by Beutner et al. [15] indicated metabolic profiling changes associated with an active lifestyle, suggesting it as a possible modulator of atherosclerosis risk, including alterations in amino acids.

Our analysis suggests a relationship between higher levels of threonine, cystine, and proline in the circulatory system and atherosclerosis in the coronary arteries, as well as the prevalence of its location in the left descending artery. The odds ratio for threonine per 1 µM increment is modest (OR 1.05); the observed range between groups suggests that clinically meaningful shifts (e.g., 50 µM) could increase CAD risk more than tenfold. A consistent conclusion can be drawn regarding the location of atherosclerotic plaques in specific coronary arteries and the presence of cystine and proline.

In previous analyses, disrupted arginine metabolism was reported to be crucial for endothelial function and vascular tone [16]. In an animal model, the immunomodulatory effect of homoarginine was noticed, suggesting its influence on atherosclerotic size [17]. The pro-inflammatory properties, combined with their potential involvement in plaque instability, were linked with dietary branched-chain amino acids (BCAAs) [18]. Sex-related differences in BCAAs concentration in relation to body mass index, including fat tissue metabolism, have been suggested in previous studies [19]. The potential impact of glutamine on macrophage function has been proposed [20]. In other analyses [21], the glycine and taurine supplementation were postulated to improve endothelial function and decrease the risk of atherosclerotic plaque formation.

Increased circulating threonine levels were found to be significant for coronary disease, including the number of involved arteries and Cx atherosclerotic involvement. Previous analyses have indicated the potential impact of this essential amino acid on ischemic heart disease, but the relationship was described as complex and not fully understood [22,23].

One of the key amino acids in cardiovascular pathology is glycine, which is involved in the folate cycle and one-carbon metabolism, and is synthesized from various precursors, including the breakdown of threonine by threonine dehydrogenase [24]. One-carbon metabolism supports mitochondrial activity, leading to increased transport chain (ETC) flux [25]. The ETC activity can result in electron leakage, inducing the formation of reactive species, such as superoxide (ROS) [26]. This is one of the possible mechanisms linking increased threonine concentration to atherosclerotic risk. Another explanation for our results can be attributed to the stimulation of mTOR and anabolic pathways by high threonine levels, leading to increased ROS formation secondary to enhanced mitochondrial activity [27]. The threonine can be transformed into acetyl-CoA and succinyl-CoA, two intermediates that promote ROS formation by NADH/FADH2 in the Krebs cycle—the fourth possible pathway. Linking higher threonine levels to increased atherosclerosis risk may be explained by their interplay with the formation of glutathione and other antioxidant systems. The imbalance in glutathione formation resulting from insufficient production may lead to the development of oxidative stress [28]. Previous analyses [29] have indicated a higher atherosclerotic risk associated with circulating amino acids, independent of age or kidney function.

Our study highlights the relationship between high cystine concentration and an increased risk of coronary disease. Cystine is gaining recognition as a sensitive indicator of vascular oxidative stress [30]. Research has shown that in healthy adults, higher plasma cystine levels are independently linked to increased arterial stiffness and poorer endothelial function [31]. These observations reinforce the idea that circulating cystine serves as a marker of oxidative damage within the vasculature. Furthermore, in patients already diagnosed with CAD, increased cystine levels, along with a higher cystine-to-glutathione ratio, have been found to predict worse clinical outcomes. Specifically, higher plasma cystine levels have been associated with a significantly increased mortality risk [31]. Similar observations have been made in high-risk groups of patients undergoing hemodialysis, where elevated cystine levels were related to both cardiovascular disease mortality and all-cause mortality [32]. These findings suggest that oxidized cystine, rather than reduced cysteine alone, plays a crucial role in the development of atherosclerosis. In our analysis, high cystine concentrations were explicitly associated with LAD atherosclerotic involvement. This novel finding suggests that the oxidative status of cysteine (reflected by cystine levels) may play a unique role in localized CAD, providing a new perspective on the interplay between thiol oxidation and atherosclerotic risk.

Our analysis suggests a relationship between circulating proline levels and an increased risk of coronary artery disease, including involvement of the left descending artery. This amino acid plays a crucial role in maintaining redox homeostasis. The ROS and ATP are generated through intracellular proline catabolism [11]. Schworer et al. [33] found that increased proline biosynthesis occurs in response to a reduction in mitochondrial redox potential. The presented results contrast with previous reports, which have shown proline to have a beneficial role in reducing post-myocardial scar, oxidative stress, and apoptosis [34]. The higher circulating levels of this non-essential amino acid, whose metabolism is regulated by redox balance and cell survival, may indicate proline derangements in CAD patients.

The role of the ROS paradigm in cardiovascular pathophysiology can be related to disease progression and adaptive changes in response to chronic myocardial ischemia. Our results suggest a potential relationship between higher serum amino acid levels and coronary artery disease risk, highlighting the involvement of specific arteries as a potential therapeutic target. Amino acids can be regarded as a possible link between ROS activation and atherosclerotic risk. In a previous report [35], the authors focused on the pathophysiology of chronic coronary artery occlusion and the role of H2O2-mediated arteriolar dilation dysregulation. The role of BKCa and Kv channels in adaptation to myocardial ischemia related to exercise training was presented in animal studies [36]. The positive effects resulting from genetic or pharmacological mitochondrial monoamine oxidase inhibition in cardiac pathologies related to mitochondrial function, cellular metabolic integrity, and viability [29]. Bertero et al. [37] highlighted the role of mitochondrial Ca2+ levels in regulating the rate of oxidative metabolism, as high calcium levels in the mitochondrial matrix ultimately led to cell death. The protective role of sodium-glucose cotransporter 2 (SGLT2) in cardiovascular diseases, including heart failure, is related to lower calcium levels in endothelial cells [38].

Increased circulatory levels of amino acids in coronary artery disease and their potential relationship to the atherosclerotic involvement of specific coronary arteries are the novelty of our analysis. The derangement of amino acid metabolism can be considered a causative factor in the development of atherosclerotic plaque formation, which warrants further investigation.

Our results suggest potentially distinct pathophysiological backgrounds of atherosclerosis formation in specific coronary arteries. The explanation for why coronary plaques are formed in particular arteries remains unsolved. Previous studies indicated the possible influence of hemodynamic differences on plaque location in the coronary bed [39]. The association between various trace element concentrations, as cofactors of distinct enzymatic cascades, may explain the particular involvement of coronary arteries in the formation of atherosclerotic plaques [40].

### Study Limitations

The study involved a relatively limited number of elderly consecutive patients presenting with chronic coronary syndrome as a single-center prospective analysis. To confirm the given hypothesis, further studies are required in the future.

The presented results were not validated in healthy individuals, but rather in a control group with normal coronary angiograms, which had comparable demographic and clinical profiles, including the prevalence of comorbidities.

The significant plaque burden, defined as coronary lumen narrowing at least 50%, was estimated based on the experienced hemodynamic team’s description of plain coronary angiograms, routinely applied in clinical practice without additional analysis, such as intravascular ultrasonography (IVUS) or optical coherence tomography (OCT). As the results were compared to normal angiograms, but not atherosclerotic plaques, with narrowing of 10–49% of the lumen, we firmly believe that further studies are advised.

The presented analysis focused on amino acid concentration differences between the analyzed groups; however, due to its exploratory character, multivariable models were created without adjustments to the full LC-MS/MS QC or the clinical and demographic characteristics, combined with comorbidities or laboratory results, such as kidney or liver function tests.

## 5. Conclusions

Patients with coronary artery disease may be characterized by increased circulating amino acids, such as cystine, threonine, methionine, and proline, according to our initial prospective study. A weak correlation was noted between the number of involved coronary arteries and the levels of threonine, methionine, and proline. The multivariable models for left descending artery disease prediction revealed higher values of two amino acid concentrations: cystine and proline. Higher concentrations of threonine and proline were found to be prognostic in multivariable models for predicting disease in the circumflex and right coronary arteries. Further studies are mandatory to confirm the suggested theory.

## Figures and Tables

**Table 1:** Demographical and clinical characteristics.

Parameters	CAD Group n = 27	Non-CAD Group n = 27	*p*
Demographics:			
Age [years] (median (Q1–Q3))	66 (62–72)	68 (61–72)	0.633
Sex (male (%)/female (%))	18 (67)/9 (33)	15 (56)/12 (44)	0.413
BMI [kg/m^2^] (median (Q1–Q3))	27.2 (25.2–30.4)	27.8 (25.9–33.5)	0.242
BMI > 30 (n (%))	7 (26)/20 (74)	9 (33)/18 (67)	0.622
Comorbidities:			
Arterial hypertension (n (%))	18 (67)	17 (63)	0.893
Diabetes. mellitus (n (%))	9 (33)	4 (15)	0.155
Dyslipidemia (n (%))	25 (93)	20 (74)	0.142
COPD (n (%))	2 (7)	3 (11)	0.582
Kidney disease * (n (%))	10 (37)	5 (19)	0.224
Family history (n (%))	3 (11)	6 (22)	0.233
Nicotine:			
Active smoking (n (%))	5 (19)	2 (7)	0.254
Former smoker (n (%))	9 (33)	7 (26)	0.621

Abbreviations: BMI—body mass index, CAD—coronary artery disease, COPD—chronic obstructive pulmonary disease, n—number, Q—quartile. * Kidney disease is defined as a glomerular filtration rate (GFR) below 60 mL/min/1.73 m^2^.

**Table 2:** Comparison of laboratory results between the CAD group and the control group.

Parameters	CAD Group n = 27	Non-CAD Group n = 27	*p*
Whole blood count			
WBC [10 × 9/dL] (median (Q1–Q3))	7.91 (6.14–10.31)	6.89 (6.44–7.93)	0.342
Hb [10 × 9/dL] (median (Q1–Q3))	9.00 (8.30–9.60)	8.95 (8.50–9.30)	0.696
Hct [%] (median (Q1–Q3))	44 (40–45)	43 (42–45)	0.643
Plt [10 × 9/dL] (median (Q1–Q3))	238 (201–266)	268 (168–321)	0.682
RDW [%] (median (Q1–Q3))	13.2 (12.9–13.6)	12.7 (12.4–13.2)	0.032
Kidney function tests:			
Serum creatinine [umol/L] (median (Q1–Q3))	91 (84–106)	74 (69–85)	0.017
GFR [mL/min/m^2^] (median (Q1–Q3))	68 (51–81)	73 (69–80)	0.144
Liver function tests:			
ALT [IU/mL] (median (Q1–Q3))	24 (21–51)	26 (21–47)	0.823
NT–pro–BNP [pg/mL] (median (Q1–Q3))	691 (402–1781)	511 (242–906)	0.352
Lipoprotein (a) [mg/dL] (median (Q1–Q3))	2.20 (1.76–4.33)	3.30 (2.43–5.10)	0.424
CK-MB [ng/mL] (median (Q1–Q3))	2.70 (1.55–2.96)	1.30 (1.00–1.80)	<0.001
Lipidogram:			
Total cholesterol [mmol/L] (median (Q1–Q3))	5.10 (3.23–6.15)	4.47 (4.07–7.17)	0.567
HDL [mmol/L] (median (Q1–Q3))	1.23 (1.12–4.23)	1.46 (1.35–1.96)	0.728
LDL [mmol/L] (median (Q1–Q3))	3.18 (1.54–4.50)	2.85 (1.88–5.13)	0.435
Triglycerides [mmol/L] (median (Q1–Q3))	1.68 (1.41–1.89)	1.72 (1.43–1.83)	0.747

Abbreviations: ALT—alanine transaminase, CK-MB—creatine kinase MB, GFR—glomerular filtration rate, Hb—hemoglobin, Hct—hematocrit, HDL—high-density lipoprotein, LDL—low-density lipoprotein, n—number, NT–pro–BNP, Plt—platelets, RDW—red cell distribution width, Q—quartile, WBC—white blood count.

**Table 3:** Circulating amino acid concentrations in CAD and control groups.

Amino Acids [uM] (Median (Q1–Q3))	LOD Empirical	LOD Analyte	CAD Group n = 27	Non-CAD Group n = 27	*p*
1-methylhistidine	0.560	0.560	7.82 (6.21–10.42)	6.21 (4.77–9.06)	0.141
Alfa-aminobutyric acid	1.415	1.415	20.75 (18.48–27.39)	22.47 (19.51–27.93)	0.562
Alanine	9.834	9.834	412 (378–514)	403 (349–445)	0.261
Allo-isoleucine	0.615	0.615	1.95 (1.39–2.60)	1.67 (1.39–2.01)	0.223
Asparganine	10.642	10.642	46.47 (45.60–56.69)	46.67 (41.03–51.47)	0.203
Glutamine	67.153	67.153	595 (555–658)	593 (519–626)	0.397
Histidine	20.440	115.657	92.02 (87.05–113.75)	99.06 (84.45–99.12)	0.416
Taurine	9.807	19.409	132 (102–167)	151 (114–164)	0.436
Beta-aminoisobutyric acid	0.170	0.170	2.67 (1.94–4.19)	2.56 (1.37–4.42)	0.500
Arginine	6.509	27.542	84.84 (76.54–97.17)	82.13 (71.41–103.25)	0.628
Aspartic acid	7.321	7.321	22.25 (18.54–27.96)	26.74 (20.56–33.23)	0.183
Ethanolamine	0.389	0.389	9.34 (8.76–10.45)	9.84 (8.31–11.25)	0.141
Glycine	124.104	124.104	250 (208–277)	256 (215–282)	0.706
Lysine	20.570	115.831	206 (171–221)	196 (172–225)	0.856
Sarcosine	1.004	1.004	1.45 (1.23–1.80)	1.40 (1.11–1.85)	0.684
Valine	28.335	28.335	247 (212–280)	246 (232–264)	0.815
Beta-Alanine	2.583	2.583	2.82 (2.43–3.36)	3.00 (2.56–4.30)	0.400
Citrulline	6.952	6.952	33.72 (29.21–42.16)	35.80 (33.36–37.35)	0.545
Cystine	3.246	15.003	58.34 (51.34–77.26)	51.84 (42.72–58.63)	0.036 *
Leucine	37.650	91.335	141 (123–159)	142 (119–146)	0.571
Serine	9.283	9.283	142 (120–167)	144 (123–155)	0.659
Threonine	17.027	17.027	127 (104–149)	99 (91–118)	0.006 *
Tyrosine	5.338	5.338	70.31 (59.52–81.03)	66.82 (60.60–76.31)	0.706
4-Hydroxyproline	0.443	0.443	11.82 (8.34–14.25)	13.53 (7.39–16.28)	0.684
Methionine	4.375	4.375	27.05 (24.39–29.16)	23.60 (20.14–26.97)	0.013 *
Phenyloalanine	4.741	4.741	76.43 (65.43–88.24)	76.16 (68.31–87.43)	0.917
Pipecolic acid	0.891	0.891	1.43 (1.10–1.93)	1.37 (0.92–1.66)	0.337
3-methylhistidine	4.045	5.370	13.39 (8.36–22.59)	11.26 (5.86–23.00)	0.595
Alpha-aminoadipic acid	0.219	0.219	1.10 (0.90–1.42)	1.06 (0.82–1.28)	0.736
Glutamic acid	23.869	23.869	73.70 (62.78–100.43)	73.24 (55.96–107.35)	0.972
Proline	16.765	16.765	192 (171–231)	173 (162–192)	0.042 *
Tryptophane	0.716	0.716	58.3 (49.61–68.74)	61.18 (52.33–68.24)	0.643

Abbreviations: CAD—coronary artery disease, LOD—limit of detection, n—number, μM—micromol, Q—quartile, * statistically significant.

## Data Availability

The datasets generated and/or analyzed during the present study are available from the corresponding author upon reasonable request.
